# Clinical risk factors and outcomes of massive ascites accumulation after discontinuation of peritoneal dialysis

**DOI:** 10.1080/0886022X.2019.1700804

**Published:** 2019-12-12

**Authors:** Chien-Liang Chen, Nai-Ching Chen, Chih-Yang Hsu, Chien-Wei Huang, Po-Tsang Lee, Kang-Ju Chou, Hua-Chang Fang, Ming-Shan Chang

**Affiliations:** a Division of Nephrology, Kaohsiung Veterans General Hospital, Kaohsiung, Taiwan; b Department of Medicine, National Yang-Ming University School of Medicine, Taipei, Taiwan; c Department of Neurology, Kaohsiung Chang Gung Memorial Hospital, Chang Gung University College of Medicine, Kaohsiung, Taiwan; d Department of Medicine, Chung Shan Medical University, Taichung City, Taiwan

**Keywords:** Ascites, encapsulating peritoneal sclerosis, peritoneal dialysis

## Abstract

**Background:**

Encapsulating peritoneal sclerosis (EPS) is a serious complication of peritoneal dialysis (PD), with high morbidity and mortality that requires an early diagnosis for effective treatment. PD withdrawal and bacterial peritonitis are important triggers for the onset of EPS. However, few studies have focused on cases of PD withdrawal without a clinical diagnosis of peritonitis, cirrhosis, or carcinomatosis. We aimed to compare the clinical characteristics and computed tomography (CT) images of patients with or without ascites in such situations and assess clinical outcomes in terms of mortality.

**Methods:**

Our retrospective review included 78 patients who withdraw PD between January 2000 and December 2017.

**Results:**

Ten patients had ascites, and 68 did not have a significant intra-abdominal collection. The ascites group had a significantly longer PD duration (months; 134.41 [range, 35.43–181.80] vs. 32.42 [733–183.47], *p* < 0.001) and higher peritoneal membrane transport status based on the dialysate-to-plasma ratios of creatinine (0.78 ± 0.08 vs. 0.68 ± 0.11, *p* = 0.009) and glucose (0.27 ± 0.07 vs. 0.636 ± 0.08, *p* = 0.001) than the control group. CT parameters, including peritoneal calcification, thickness, bowel tethering, or bowel dilatation, were not all present in each patient with ascites and EPS. During the 12-month study period, the ascites group had a higher risk for developing EPS (70% vs. 0%, *p* < 0.001) and a higher 12-month all-cause mortality (30% vs. 0%, *p* = 0.002).

**Conclusions:**

Ascites accumulation was not rare after PD discontinuation. A longer PD duration and high peritoneal membrane transport status could predict subsequent ascites accumulation. Furthermore, patients with ascites were at a higher risk of EPS.

## Introduction

Encapsulating peritoneal sclerosis (EPS) is a progressive inflammatory condition that leads to peritoneal membrane fibrosis and adhesion, with the most serious complication of intestinal obstruction and malnutrition [1–3]. The high morbidity and mortality associated with EPS make it the most serious complication associated with peritoneal dialysis (PD). Mortality of up to 100% has been reported in patients receiving PD for >15 years [4]. According to the ‘two-hit’ theory put forward by Honda and Oda [5], withdrawal of PD (the first hit) and bacterial peritonitis (the second hit) are important triggers for the onset of EPS. Their theory is supported by the fact that 63% of EPS cases develop within 1 year of PD withdrawal [6]. Some data on PD with refractory peritonitis have been reported [7,8]. Unresolved intra-abdominal infection is associated with adhesions. However, limited data are available on patients with PD withdrawal not due to refractory peritonitis.

Patients who have undergone PD withdrawal are therefore in a unique clinical situation and are at risk of developing EPS. The challenge in managing EPS lies in early diagnosis. In 2005, Nakamoto [9] proposed a staging system for EPS, including the pre-EPS, inflammatory, encapsulating, and ileus phases. However, the clinical features in the early (inflammatory) phase are nonspecific, which frequently leads to underdiagnosis. Most available studies refer to a definite diagnosis of EPS when intestinal obstruction has already occurred, which is indeed a very late feature [9–12]. Identifying patients during the early inflammatory phase when they are at a risk of progression to full-blown EPS is therefore of paramount importance to inhibit disease progression and improve outcomes [13]. According to a previous study, cases with massive ascites accumulation after PD discontinuation are related to the concept of a pre-encapsulating peritoneal sclerosis (pre-EPS) state [14]. Ascites is an important diagnostic clue that indicates the development of EPS after PD suspension. We conducted a retrospective study to identify the clinical risk factors of patients with or without symptomatic ascites after PD withdrawal and the indication to discontinue PD other than refractory peritonitis and to compare patients’ clinical characteristics and outcomes, including short-term all-cause mortality and development of full-blown EPS.

## Methods

The study was approved by the Institutional Review Board of Kaohsiung Veterans General Hospital, Kaohsiung, Taiwan (protocol title: Clinical risk factors for patients with massive ascites accumulation after discontinuation of peritoneal ascites, VGHKS 18-CT7-17). This single-center retrospective study included all patients who underwent PD withdrawal because of poor clearance, ultrafiltration failure, or social medical problems between January 2000 and December 2017 to identify the clinical risk factors of patients with or without symptomatic ascites and to compare their clinical characteristics and outcomes, including short-term all-cause mortality and development of full-blown EPS. As with some studies that indicated that beta-blockers or RAS inhibitors can affect the development of EPS [15–17], this study included possible clinical risk factors. Patients who discontinued PD because of refractory peritonitis were excluded. We deliberately excluded patients with cirrhosis, peritoneal carcinomatosis, and ongoing bacterial/tuberculosis/fungus peritonitis/intra-abdominal sepsis from this study, as these diseases are well known to represent a different pathophysiological mechanism and carry a different prognosis. The International Society for Peritoneal Dialysis diagnostic criteria were used, which includes at least 2 of the following criteria: abdominal pain or cloudy PD effluent, leukocytosis in dialysate (>100/μL), and positive Gram stain or dialysate culture [18]. The peritoneal equilibration test, performed as described previously [19], was used to estimate solute transport. The patients underwent the test once a year. Peritoneum clearance is quantified by referring to the kinetics of urea nitrogen (UN) and creatinine (Cr) clearance, expressed weekly CCR (creatinine clearance) every 6 months. Briefly, a standard 4-h dwell period was used (first exchange of the day), with a 2.5% glucose concentration and 2-L volume exchange. The patients used their usual overnight dialysis regimen, and both the overnight and test drainage volumes were measured. The dialysate-to-plasma ratio of creatinine (D/P-crea) at the completion of the 4-h dwell period is an estimate of low-molecular-weight solute transport. Considering that glucose interferes with the assay for creatinine linearly, the concentrations of both solutes were measured at 4 h, and the true creatinine concentration was obtained by subtracting the glucose concentration multiplied by a correction factor of 0.00005 (derived locally by our laboratory). By using this method, 4-h D/P-crea is a highly reproducible measure of the low-molecular-weight solute transport across a wide range of values (0.45–0.9).

For patients with significant intra-abdominal collections, image-guided abdominal tapping (both diagnostic and therapeutic) was routinely performed when possible to rule out ongoing peritonitis, peritoneal carcinomatosis, or cirrhosis of the liver. For the study patients, parameters were collected, and their clinical courses were followed up for 12 months post PD discontinuation. Baseline demographic and clinical data, including age, sex, underlying renal disease, mode and duration of PD, and the total number of peritonitis episodes, were recorded. Outcome measures, including the occurrence of full-blown EPS (clinically presenting with nausea and vomiting, and defined as the presence of intestinal obstruction with features of bowel encapsulation on computed tomography [CT] scans), during the study period and all-cause mortality (12 months) post PD discontinuation were assessed.

### CT scans

CT scans of the abdomen and pelvis were included in our study. Only patients who had additional CT scans taken before and/or after PD discontinuation after 6 months were included the studies. CT scans were viewed on PACS workstations and considered to be of diagnostic quality by the radiologists involved. No clinical information was revealed to the radiologists at the time of the assessments. Abdominal CT scans (Sensation 16, Siemens, Forchheim, Germany; parameters: SD, 5 mm; increment, 5 mm; 120 kV at 217 mAs) were performed at Kaohsiung Veterans General Hospital. Scoring parameters were given on the basis of previously published data on CT abnormalities found in EPS [20,21]. The abdominal CT parameters, including peritoneal calcification (0–4), peritoneal thickening (0–4), bowel tethering (0–3), and bowel dilatation scores (0–4), were recorded [20].

## Statistical analyses

The statistical analysis was performed using the SPSS version 13.0 software application (SPSS, Chicago, IL, USA). Continuous data are expressed as mean ± standard deviation or median (range) unless otherwise specified, and categorical data are expressed as number and percentage. The differences between the patients with and without ascites were analyzed using the chi-square or Fisher exact test for categorical data, as appropriate; *t* test for continuous normally distributed data; and Mann-Whitney test for non-normally distributed data. Variables with a *v*alue of <0.1 in the univariate analyses were included in the multivariate binary logistic regression analysis. Receiver-operating characteristic (ROC) analysis, with an estimate of the area under the ROC curve (AUC), was used to identify significant predictors of ascites formation. A *p* value of <0.05 was considered statistically significant. Multivariate logistic regression analyses were performed using all the variables that could be used to investigate the factors associated with EPS.

## Results

**Figure 1.**
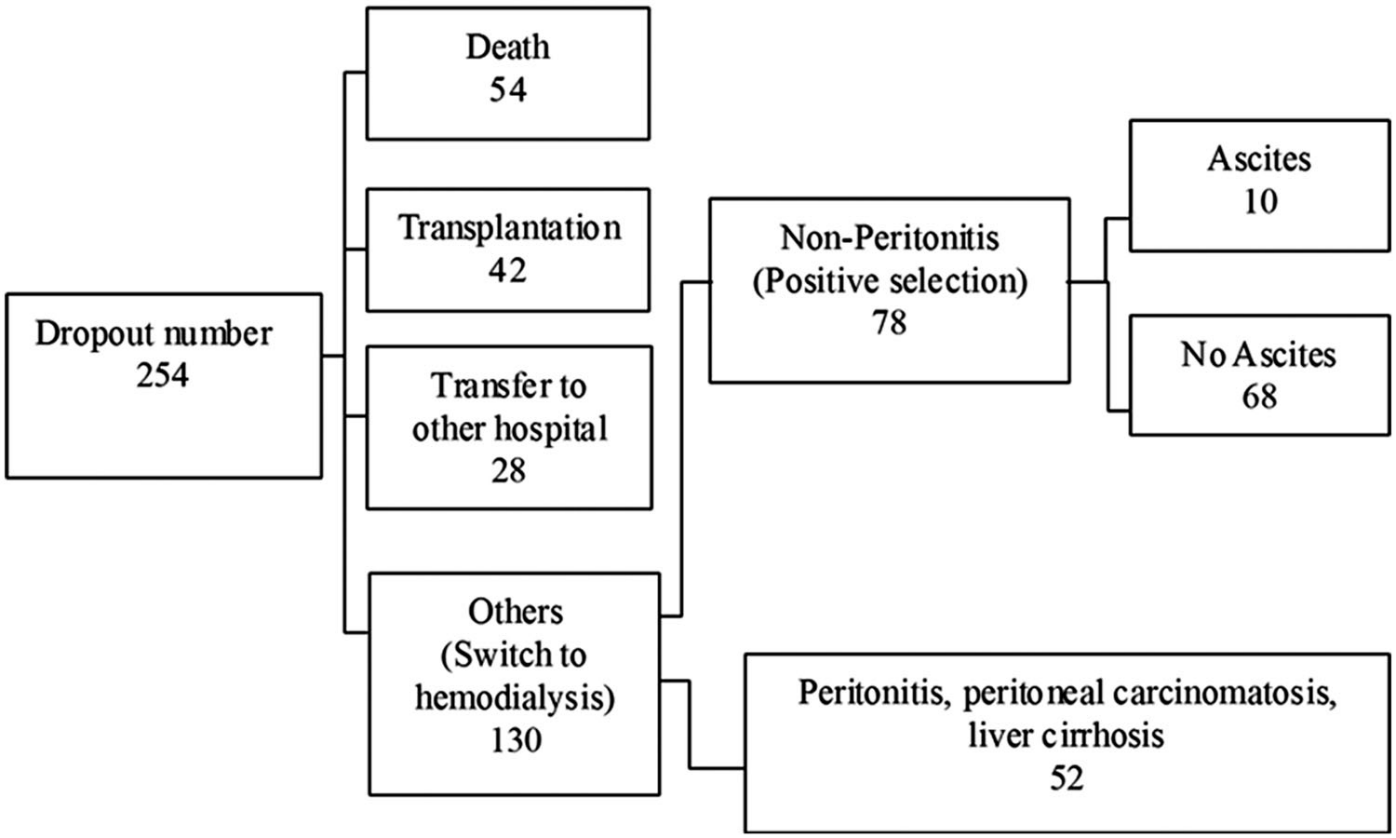
Study process flowchart.

**Figure 2.**
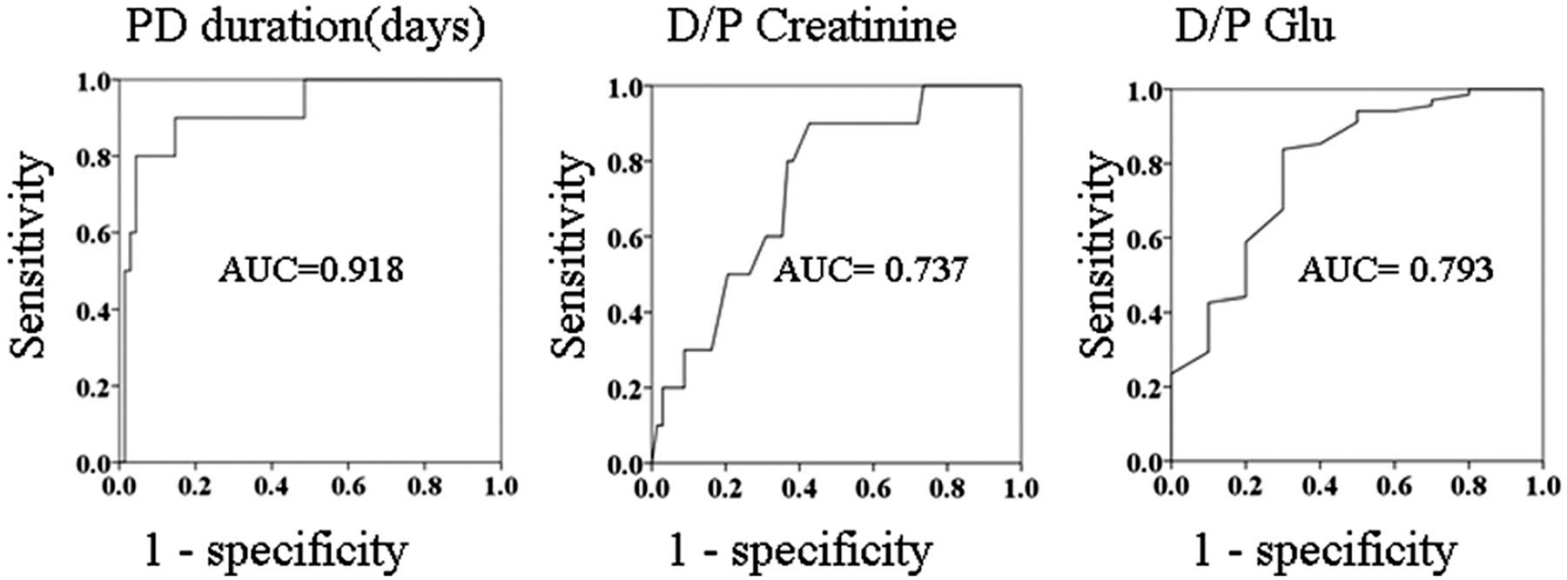
(a) Receiver-operating characteristic (ROC) curve of the time to ascites development. Cutoff point, 70.74 months (sensitivity: 90% and specificity: 85.3%). (b) ROC curve of dialysate-to-plasma creatinine ratio for ascites development. Cutoff point, 0.705 (sensitivity 90.0% and specificity: 57.4%). (c) ROC curve of dialysate-to-plasma glucose ratio without ascites development. Cutoff point, 0.335 (sensitivity: 58.8% and specificity: 80.0%).

After exclusion of the patients who discontinued PD because of transfer to another hospital, death, or peritoneal catheter removal related to refractory peritonitis, only 78 patients were enrolled in this study (Figure 1). Their clinical data are shown in Table 1. The baseline characteristics of the patients with ascites were not similar to those of the patients without ascites (Table 2). Among the 78 patients included in this study, 10 developed persistent peritoneal ascites. Compared with the control group, the ascites group had a significantly longer PD duration (134.41 months [range, 35.43–181.80 months] vs. 32.42 months [7.33–183.47 months], *p <* 0.001), a higher peritoneal membrane transport status as evidenced by the dialysate-to-plasma ratios (D/P) of creatinine (0.77 ± 0.09 vs. 0.69 ± 0.11, *p* = 0.014) and glucose (0.27 ± 0.08 vs. 0.36 ± 0.08, *p* = 0.001). There are significant differences in PD duration (*p* < 0.01), D/P-crea (*p* < 0.05), and higher peritoneal membrane transport status (*p* < 0.05). On the receiver operating characteristic (ROC) curves, the cutoff points for ascites were a PD duration of 74.74 months and D/P-crea of 0.705 and the cutoff point without ascites was a D/P-glu of 0.335 (Figure 2(a–c)). As shown in Table 3, the multivariate analysis revealed that PD duration was the only independent risk factor for predicting ascites formation after PD discontinuation. The results showed that the presence of 2 of the risk factors (PD duration of >6 years and D/P-crea >0.705) had better results with good discriminability (AUC: 0.879, *p* < 0.001; sensitivity: 0.909 and specificity: 0.881).

**Table 1.** Patients’ demographic data.

N	78
Sex (male/female)	40/38
Age (years)	51.53 ± 14.79
PD duration (days)	1669.59 ± 1411.99
D/P-crea	0.70 ± 0.11
D/P-glu	0.35 ± 0.85
Higher transporter (>0.705)	38 (48.72%)
Ultrafiltration failure	10 (12.82%)
Incidence of ascites	11 (14.10%)
Reason for change from PD to HD	
Social problem	14 (17.95%)
Intra-abdominal surgery	8 (10.26%)
Poor clearance	42 (53.85%)
Ultrafiltration failure	10(12.82%)
Tunnel infection	4 (5.13%)
Cause of CRF	
DM	17
CIN	17
CGN	33
Lupus nephritis	6
Hypertensive nephrosclerosis	1
Other/unknown	4

PD: peritoneal dialysis; HD: hemodialysis; CRF: chronic renal failure; DM: diabetes mellitus; CIN: chronic interstitial nephritis; CGN: chronic glomerulopathy; D/P-crea: dialysate-to-plasma creatinine ratio.

The values presented as mean ± SD or n (%).

**Table 2.** Demographic data of the patients with and without ascites.

	With ascites	Without ascites	*p* Value
Number of patients	10	68	
Sex (female/male)	4/6	34/34	0.738
Age (years)*	49 (18–55)	54.5 (13–81)	0.036
PD duration (months)*	134.41 (35.43–181.80)	32.42 (7.33–183.47)	<0.001
Weekly creatinine clearance	58.95 ± 8.37	64.03 ± 40.79	0.59
Ultrafiltration of the peritoneal equilibration test (ml)	72.22 ± 158.33	141.15 ± 180.91	0.14
D/P-crea*	0.77 ± 0.09	0.69 ± 0.11	0.018
D/P-glu*	0.27 ± 0.08	0.36 ± 0.08	0.001
Higher transporter status (D/P-crea > 0.705)*	9 (90.0%)	32(47.1%)	0.006
Total number of peritonitis episodes	0.60 ± 1.07	0.13 ± 0.38	0.227
Incidence of peritonitis (n/years)	0.05 ± 0.08	0.04 ± 0.15	0.926
History of ACEI or ARB use	3 (30%)	25 (36.8%)	1.0
History of beta-blocker use	2 (20%)	20 (29.4%)	0.716
Reason for change from PD to HD			
Tunnel infection	0	4 (5.88%)	1.000
Poor clearance	5 (50.00%)	37 (54.41%)	0.500
Ultrafiltration failure	2 (20.00%)	8 (11.76%)	0.608
Other/social problem	4 (40.00%)	18 (26.47%)	0.455
Cause of CRF			
DM	0	17 (25%)	0.11
CIN	2 (20%)	15 (22.06%)	1.0
CGN	6 (60%)	34 (50%)	0.74
HTN	1 (10%)	0	0.13
Other/unknown	1 (10%	2 (2.94%)	0.34
Outcome			
Full-brown sclerosing peritonitis*	7 (70%)	0	<0.001

The values presented are mean ± SD, median (range), or n (%).

PD: peritoneal dialysis; HD: hemodialysis; CRF: chronic renal failure; DM: diabetes mellitus; CIN: chronic interstitial nephritis; CGN: chronic glomerulopathy; D/P-crea: dialysate-to-plasma creatinine ratio; ACEI: angiotensin-converting enzyme; ARB: angiotensin receptor blocker.

*p < 0.05.

**Table 3.** Multivariate analysis of the risk factors for ascites development after peritoneal dialysis discontinuation.

Variable	Odds ratio^a^ 95% CI	Odds ratio^b^ 95% CI	Odds ratio^c^ 95% CI
Age	0.976 (0.919–1.035)	0.954 (0.909–1.001)	0.982 (0.922–1.046).
PD duration (>72 months) (<72 months = 1 as reference)	40.726 (4.617–359.275)*	–	26.117 (2.523–270.365)*
Higher transporter status (D/P-crea > 0.705) (D/P-crea <0.705 = 1 as reference)	–	14.503 (1.631–128.952)*	2.979 (0.256–34.611)

^a^Model 1: age and PD duration.

^b^Model 2: age and higher peritoneal membrane transports.

^c^Model 3: age, PD duration, and peritoneal membrane transport.

*p < 0.05.

Abdominal CT scans from the time of ascites diagnosis were available in 10 patients with ascites and 26 patients without ascites. Some patients with peritoneal calcification and peritoneal thickening did not have ascites. Minor abnormalities were found in the 26 patients without ascites, including peritoneal calcification (1 case), peritoneal thickness (10 cases), and mild localized bowel dilation (6 cases). None of the 26 patients had evidence of bowel tethering. The CT indications for the patients without ascites included change in bowel habit, renal cell carcinoma, and acute abdomen pain. Abnormalities were found in 10 patients with ascites, including peritoneal calcification (7 cases), peritoneal thickness (9 cases), bowel tethering (8 cases), and localized bowel dilation (7 cases). The abdominal CT parameters, including peritoneal calcification, peritoneal thickness, or bowel tethering or dilatation, were not present in the patients with ascites. However, significant differences in CT parameter scores (peritoneal calcification, *p <* 0.01; peritoneal thickening*, p <* 0.01; bowel tethering, *p <* 0.01; bowel dilatation, *p <* 0.01) were found between the patients with and without ascites (Figure 4). Furthermore, abnormalities were found in seven patients with EPS, including peritoneal calcification (six cases), peritoneal thickness (seven cases), bowel tethering (seven cases), and localized bowel dilation (six cases).

**Figure 3.**
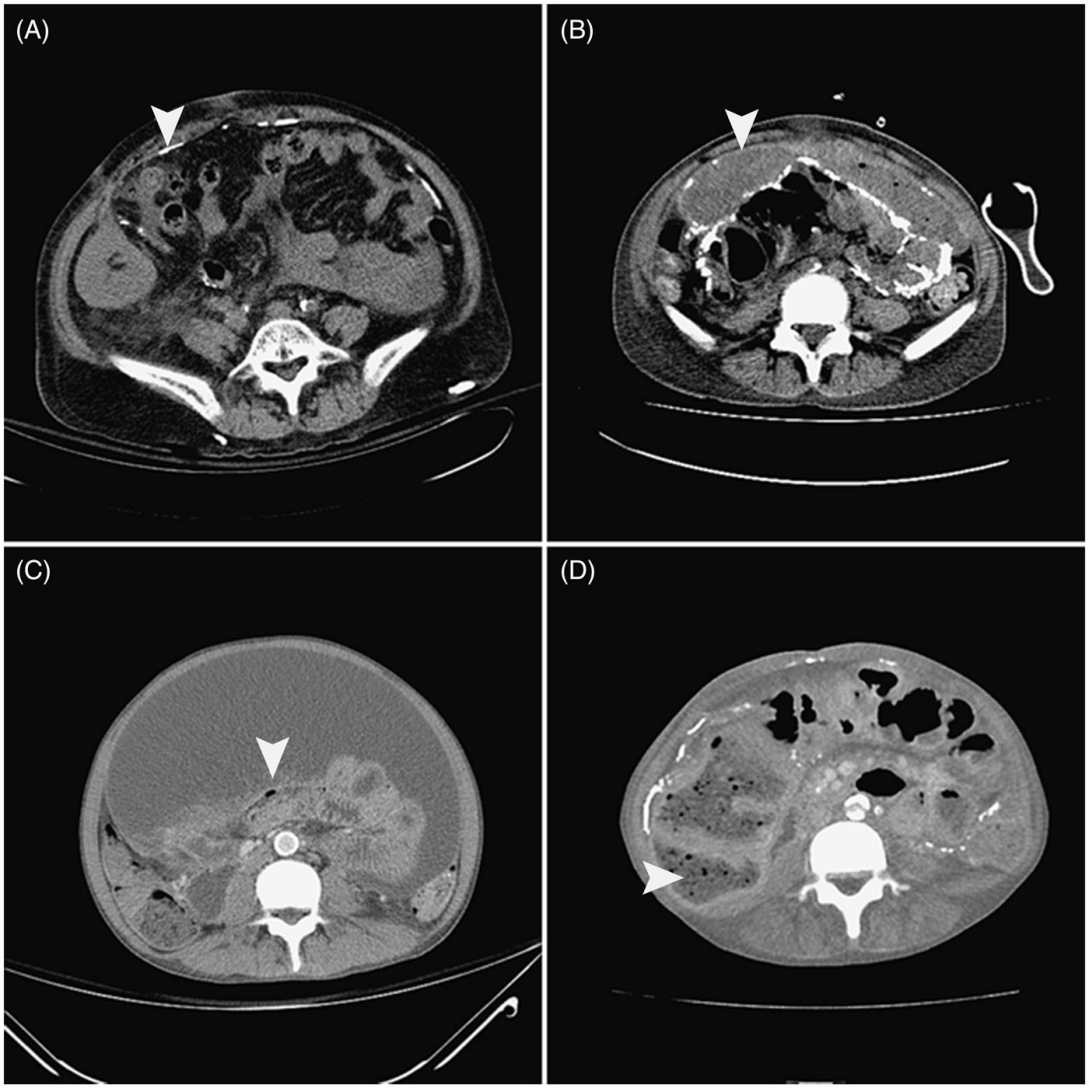
Cross-sectional abdominal computed tomography images of patients diagnosed with EPS. Note the peritoneal calcification (A, arrow), peritoneal thickening surrounding the ascites (B, arrow), bowel tethering (C, arrow), and bowel dilation (D, arrow) in the patients who discontinued peritoneal dialysis.

As shown in Table 4, in the ascites group, 7 of the 10 patients developed full-blown EPS, while none of the patients in the non-ascites group developed EPS (70% vs. 0%, *p* < 0.001). The mean time from removal of the dialysis catheter to the development of intestinal obstruction that led to the diagnosis of full-blown EPS was 72.91 ± 53.40 days (range, 6–161 days). The diagnosis of EPS was made on clinical grounds, supported by imaging investigations. The typical CT scan features of the seven patients with EPS are shown in Figure 3. Seven of the 10 patients exhibited at least one of the gastrointestinal symptoms (e.g., vomiting, nausea, loss of appetite, abdominal distension, and diarrhea) that were consistent with the clinical manifestations of early-phase EPS. Repeated therapeutic paracentesis was required for nine patients. We observed no complications related to the procedures. All the specimens were initially clear ascites and were found to be negative upon culture. However, the subsequent clinical course of the five patients without a PD catheter showed an ascites-positive culture that required antibiotic treatment. Five of the patients with ascites required long-term total parenteral nutrition, and 3 patients with ascites (30%) died of EPS-related complications such as cachexia and sepsis. The other 70% had several episodes of subacute bowel obstruction and/or blood-stained ascites, which improved after several months or years and now require only oral nutritional supplements or no nutritional support. Fixed-time all-cause mortality at 12 months was calculated for the patients from the point of PD discontinuation. The ascites group had a significantly higher 12-month all-cause mortality (30% vs. 0%, *p* < 0.05) than the non-ascites group. The causes of mortality were cachexia, poor intake, weakness, bedridden status, and later sepsis.

**Table 4.** Treatments and outcomes of patients with and without ascites.

Outcome		With ascites	Without ascites	*p* Value
n		10	68	
Confirmed EPS diagnosis		7/10	0/68	<0.001
Treatment				
Steroid		7	0	<0.001
Tamoxifen		7	0	<0.001
Outcome				
Mortality related to gastrointestinal problem <12 months		n = 3 (30.00%)	n = 0 (0%)	0.002
Cause of mortality		Cachexia and sepsis		

## Discussion

This retrospective study was conducted to clarify the clinical risk factors related to the development of ascites in 78 patients who received PD treatment. In this study, we found that persistent sterile ascites was common (14.10%) in patients without evidence of peritonitis after PD discontinuation due to ultrafiltration failure, poor clearance, and mechanical problems. Significant differences in PD duration and higher membrane transport status were observed between the patients who developed ascites and those who did not. Multivariate analysis revealed that PD duration was significant for ascites development. Only patients with ascites had an increased risk of developing full-blown EPS and had poor short-term survival, especially in the patients who had received PD for >6 years and had a high transport membrane state.

The current challenge in managing EPS is the lack of specific diagnostic criteria in the early phase, which is probably the only phase during which medical therapy can be effective. Despite medical advances in proteomics, such as tests for matrix metalloproteinase 2, its application in the diagnosis of EPS remains inconclusive [22]. Once full-blown EPS complicated with intestinal obstruction occurs, the mortality and probability of medical therapy failure are high. Surgical intervention carries a high risk of mortality. Clinical judgment is still required to identify high-risk patients suitable to undergo such investigations. According to the ‘two-hit’ theory of Honda and Oda, patients undergoing dialysis catheter removal due to refractory bacterial peritonitis have two important ‘second hit’ factors (bacterial peritonitis and withdrawal of dialysis) that predispose them to the development of EPS. Clinically, 38% of EPS cases developed after an episode of bacterial peritonitis [23], and 63% occurred after PD withdrawal for various reasons [6]. Although bacterial peritonitis [4,23] and PD withdrawal [4,24] both appear to be associated with the development of EPS, patients without peritonitis still maintain predisposing factors for the development of EPS. Despite the huge discrepancy between the control and study groups, our study still provides clinical clues. Among the overall study population of 78 patients who underwent PD discontinuation without refractory peritonitis, 14.10% developed EPS. Other than this seemingly high proportion, we could not identify any specific additional peritonitis-related risk factors, as the incidence of peritonitis (number of episodes/year) was not identified as a significant factor in the analysis. Elevated C-reactive protein level and the presence of intra-abdominal collections have commonly been observed in patients who experienced technique failure [7,8,25]. These features are likely the clinical manifestations of peritoneum inflammation. The accelerated fibrin production triggered by peritoneal sterile inflammation, if not continuously removed by PD (owing to treatment withdrawal), will rapidly deposit and encapsulate the bowel, causing bowel dysfunction. In our study, a significantly longer PD duration and higher membrane transport status were observed in the ascites group, which are findings that have been consistently identified as risk factors of EPS. Prolonged exposure to hypertonic glucose solution leads to the development of a high transport status, which is an evidence of peritoneal deterioration (i.e., the ‘first hit’), associated with a trend of higher technique failure such as that which leads to the development of ascites and later EPS even without peritonitis [26]. We conclude that the massive ascites accumulation after PD discontinuation could be regarded as a sign of pre-EPS status, and initiation of prophylactic treatment should be considered at this stage of the disease. However, peritoneal biopsy in these patients is useful for ruling out/in other serious causes of ascites with the same presentation and different diagnosis such as extramedullary plasmacytoma [27].

The peritoneal membrane with an increased transport status for solutes (small-to-macro molecules) associated with altered vasculature (e.g., vasculopathy, neoangiogenesis, and immature vasculogenesis), which occurs with long-term PD or peritoneal inflammation, could be an early marker of EPS with ascites. Patients who discontinue PD have high risks of, for example, long PD duration and high peritoneal transport status (D/P-crea >0.705); thus, in these patients, features of peritoneal inflammation should be actively monitored after the shift from PD to hemodialysis, catheter removal must be delayed if possible, and peritoneal lavage must be continued to remove inflammatory mediators to decrease the presence of intra-abdominal collections and later EPS. However, the clinical benefit of peritoneal lavage, which is the common practice in Japan, has not been elucidated in a well-designed study [28,29].

Our ROC analysis revealed that a D/P-crea of 0.705, D/P-glu 0.335, and PD duration of 74.74 months were highly sensitive and specific predictors of ascites development in the patients in this study. Our results show that the presence of two of the risk factors (PD duration > 6 years and D/P-crea > 0.705) had better results with good discriminability (AUC: 0.879, *p* < 0.001; sensitivity: 0.909 and specificity: 0.881). Although these data were the result of the univariate analysis, a combination of these parameters could be used as a marker to prevent ascites formation in clinical practice.

**Figure 4.**
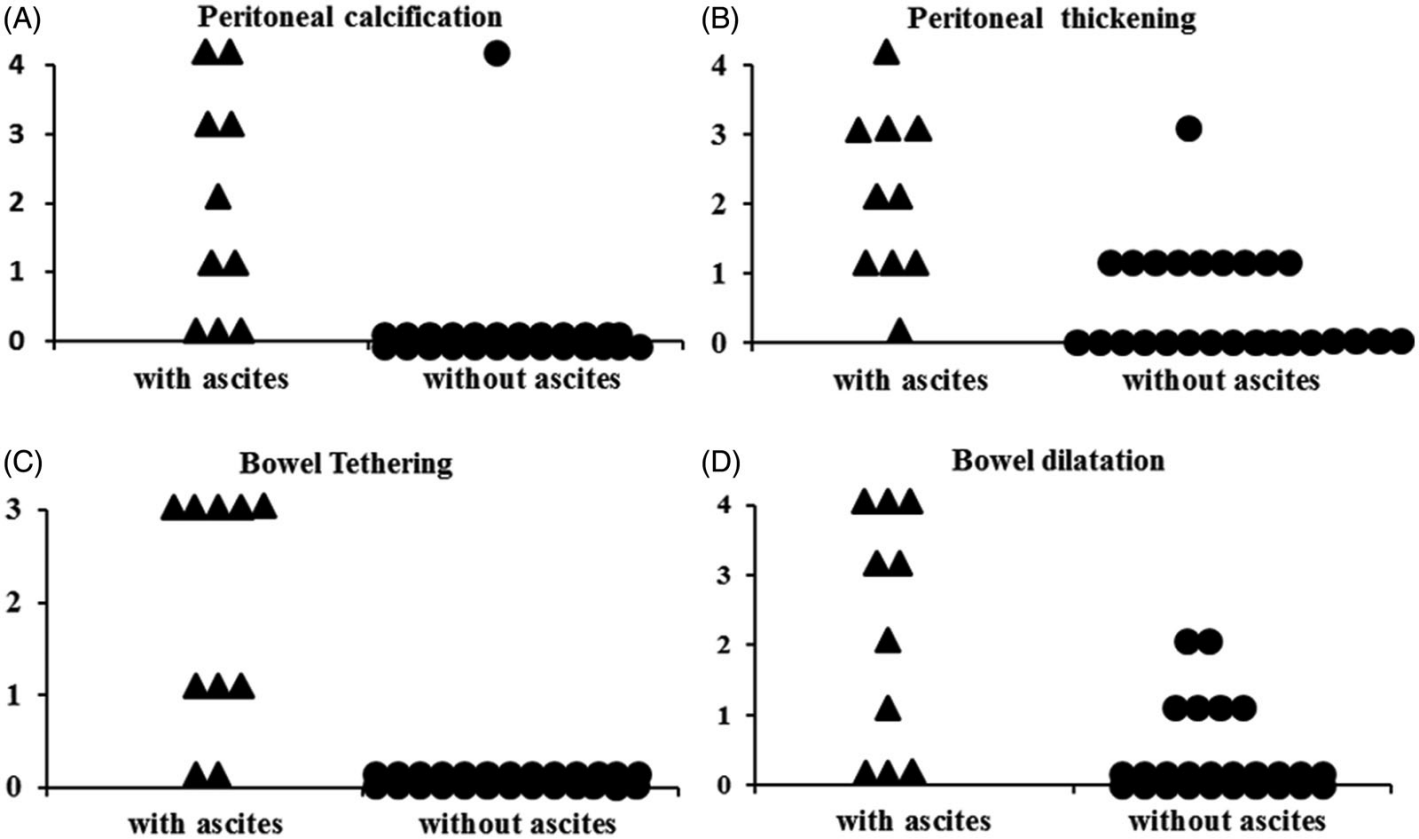
Computed tomography scores of patients with ascites and without ascites. (A) Peritoneal calcification; (B) peritoneal thickening; (C) bowel tethering; (D) bowel dilation.

Having determined which CT scan changes are suggestive of EPS [20,21], we found that the CT scan scores at the time of diagnosis of ascites showed some evidence of EPS in this study (Figure 4). Some patients with peritoneal calcification and peritoneal thickening did not have ascites. The abdominal CT parameters, including peritoneal calcification, peritoneal thickness, or bowel tethering or dilatation, were not all present in each patient with ascites. This might indicate that routine screening for PD patients using CT scans may be less useful, but CT scanning could be used as an adjunct diagnostic modality for ascites complicated with EPS. The clinical index of suspicion for EPS should be most important after PD cessation. Ascites with peritoneal thickening and calcification could be an early sign of peritoneal inflammation with the potential for developing mesentery tethering and later intestinal obstruction.

This study has a few limitations. First, we reported only patients who discontinued dialysis. However, the patients in our center developed ascites later (i.e., months after) after technical failure or poor clearance related to PD withdrawal with conversion to hemodialysis. The findings in the present study might therefore not be applicable to groups of patients who develop ascites with a preceding episode of refractory peritonitis that required catheter removal. Second, this is a single-center study involving a small number of patients of Chinese ethnicity and might not be applicable to other ethnic groups. Finally, this is a retrospective study, so the CT scanning protocols used were considered clinical indications rather than screening procedures. We found significantly more abnormalities in the CT scans of the patients with and without ascites. This needs to be confirmed in prospective studies because of the limitations of the present study due to its retrospective design and limited number of patients.

In conclusion, the present study confirmed that ascites accumulation was common after PD withdrawal in patients undergoing long-term PD (for indications other than refractory bacterial peritonitis) and that the patients with ascites were at a high risk of progression to full-blown EPS. The presence of a long PD duration and high peritoneal membrane transport status could predict ascites accumulation after PD discontinuation. Clinical suspicion EPS and the use of CT scanning as an adjunct diagnostic modality should be most important after PD cessation.
